# M. Piorry on a Peculiar Granular Appearance of the Buffy Coat of the Blood

**Published:** 1842-11-05

**Authors:** 


*Peculiar granular appearance of the buffy coat of the Blood.* By M. Piorry. In a recent lecture at the Hospital of la Pitié, M. Piorry called the attention of his class to a granular condition of the buffy coat of the blood, which he has observed only in cases in which pus was contained in some organ in considerable quantity, especially in the lungs. In these cases the buffy coat covering the blood contains rounded, grayish granulations, of a darker colour at the centre than at the circumference, varying from the size of a poppy to that of a hempseed. Sometimes five or six of these granulations are contained within the space of a square inch ; at other times they are much more numerous. They are semi-transparent at their borders and opaque at their centre. They are situated at different depths in the substance of the buffy coat, from which they can be detached with the point of a scalpel, though not easily. The buffy coat does not present any peculiar appearance, and these granular bodies have never been observed in the clot.

This appearance is far from common : M. Piorry has not met with it above twenty times. In seventeen or eighteen of these twenty cases death took place ; and in every instance pus was found in the substance of some organ, especially of the lung, which, in fifteen cases, was in a state of purulent infiltration.

Pneumonia with purulent infiltration of the lung existed in the patient whose case gave rise to the above remarks. The granulations are regarded by M. Piorry as collections of purulent matter ; and he replies to M. Donné, who denies their resemblance, under the microscope, to the true pus-globule, that the circulation of pus in the blood must alter the characters of the former, and that the results of microscopic investigations are not of value sufficient to overturn facts gathered by clinical observation.—*Brit. and For. Med. Rev. from Gaz. des Hôp.*, Avril 16, 1842.

